# Pradofloxacin Minimum Inhibitory Concentration Profiling of *Streptococcus suis* Isolates: Insights into Antimicrobial Susceptibility in Swine

**DOI:** 10.3390/pathogens14010088

**Published:** 2025-01-17

**Authors:** Jessica Risser, Ronald Tessman, Don Bade, Orhan Sahin, Maria J. Clavijo, Saumya Dhup, Patrick Hoffmann

**Affiliations:** 1 Elanco Animal Health, Greenfield, IN 46140, USA; 2 Microbial Research Incorporated, Fort Collins, CO 80525, USA; 3 Veterinary Diagnostic and Production Animal Medicine, College of Veterinary Medicine, Iowa State University, Ames, IA 50011, USAmclavijo@iastate.edu (M.J.C.); 4 Elanco Innovation and Alliance Centre, Bangalore 560008, India; saumya.dhup@elancoah.com

**Keywords:** pradofloxacin, swine, *Streptococcus suis*, respiratory disease, meningitis, minimum inhibitory concentration

## Abstract

This study evaluated the minimum inhibitory concentration (MIC) of pradofloxacin against various swine respiratory pathogens, including *Bordetella bronchiseptica*, *Glaesserella parasuis*, *Mycoplasma hyopneumoniae*, *Pasteurella multocida*, and *Streptococcus suis* (*S. suis*), associated with disease in swine. This research was conducted in two phases: the initial phase examined isolates from the lungs that could be either commensal or pathogenic, while the second phase focused on systemic *S. suis* strains that spread from the respiratory tract to the brain. The pradofloxacin MIC values of the second phase were within the MIC range of the initial phase, with MIC_50_ and MIC_90_ values highlighting its potential as an effective antimicrobial agent. Quality control data validated the reliability of our MIC findings, with all pradofloxacin MIC values for control organisms within approved ranges. These findings suggest that pradofloxacin has broad-spectrum activity against Gram-positive and Gram-negative bacteria and may serve as a reliable therapeutic option for managing *S. suis* and other swine respiratory infections. This study highlights pradofloxacin as an alternative antimicrobial therapy for swine respiratory diseases, offering a potential solution amidst rising concerns over antibiotic resistance.

## 1. Introduction

*Streptococcus suis* (*S. suis*) is a significant zoonotic pathogen in the swine industry, causing a wide range of diseases in pigs, including bronchopneumonia, meningitis, septicemia, and arthritis, leading to substantial economic losses worldwide [1,2,3]. The commensal Gram-positive bacterium can be found in the respiratory, gastrointestinal, and reproductive tracts of healthy pigs [4]. Pathogenic *S. suis* and other microorganisms establish a niche within the upper respiratory tract, specifically in the nasal cavities and tonsils of pigs [5]. Vertical transmission is common from sow to piglet during birth. Additionally, transmission can occur through close contact between pigs, such as nose-to-nose contact or via contaminated environments. The bacterium poses not only a threat to swine health, but also to public health, as it can be transmitted to humans, particularly individuals in close contact with infected pigs or pork-derived products, resulting in zoonotic infections [6].

Despite efforts to control septicemic *S. suis* on swine farms, specific treatments that target infections remain elusive, highlighting the ongoing need for effective antimicrobial agents [7]. Penicillin has traditionally been a mainstay in the treatment and control of *S. suis* infections; however, the emergence of penicillin-resistant strains necessitates the exploration of alternative therapeutic treatment options to combat infections effectively [8,9]. Previous studies evaluated the pathogenicity of *S. suis* serotype 2 in swine [10,11]. These studies underscored the pathogenicity of *S. suis* and the potential ramifications of untreated infections. This study also emphasized the pivotal role of effective antimicrobial therapy in managing *S. suis* infections in pigs.

Pradofloxacin, Pradalex^TM^, a third-generation fluoroquinolone, is approved in the United States for the treatment of *S. suis* respiratory infections in growing swine [12]. Fluoroquinolones exert their bactericidal effect by inhibiting bacterial DNA gyrase and topoisomerase IV, essential enzymes involved in DNA replication, repair, and recombination [13]. Pradofloxacin, with simultaneous effect on both enzymes, exhibits broad-spectrum activity against Gram-positive and Gram-negative bacteria, including many pathogens associated with swine respiratory diseases [14]. Its unique chemical structure and mode of action contribute to its efficacy against antibiotic-resistant bacterial strains [15]. Since resistance to pradofloxacin would require simultaneous mutations in both the DNA gyrase and topoisomerase IV enzymes, in *S. suis*, a reduced development of resistance can be expected [16,17]. There are additional ways in which fluoroquinolone resistance can develop that are unrelated to the site of action, including efflux pumps, which would likely affect pradofloxacin susceptibility. Efflux-mediated resistance to fluoroquinolones has been identified in several Gram-negative and -positive bacteria. However, this mechanism is not substrate-specific and would confer resistance to other classes of antibiotics as well as an efflux of detergents, disinfectants, and organic solvents. Further, these efflux systems are chromosomally present in both fluoroquinolone-resistant and -non-resistant bacterial isolates, suggesting a well-conserved general bacterial survival mechanism that does not necessarily require previous fluoroquinolone exposure [18,19].

In veterinary medicine, antimicrobial susceptibility testing (AST) data play a crucial role in predicting the clinical outcome of antimicrobial treatment, enabling veterinarians to make informed decisions about the most appropriate drugs to combat bacterial infections [20,21]. Typically, antimicrobial susceptibility is assessed through minimum inhibitory concentration (MIC), which represents the lowest concentration of an antimicrobial agent that inhibits visible bacterial growth in vitro and serves as one of the key parameters in determining the in vivo susceptibility of bacterial strains to specific antibiotics [22]. Determining the MIC of pradofloxacin against *S. suis* isolates is essential for assessing its antimicrobial activity and guiding treatment decisions. Knowledge of pradofloxacin’s MIC values against *S. suis* isolates linked to swine diseases is essential for guiding therapeutic protocols and mitigating the development of antibiotic resistance.

This study is divided into two phases, with the initial phase objective to determine the MIC of pradofloxacin against swine respiratory pathogens (*Bordetella bronchiseptica* (*B. bronchiseptica*), *Glaesserella* (*Haemophilus*) *parasuis* (*G. parasuis*), *Mycoplasma hyopneumoniae* (*M. hyopneumoniae*), *Pasteurella multocida* (*P. multocida*), and *Streptococcus suis* (*S. suis*)) originating from the lungs. The objective of the second phase focuses on determining the MIC of pradofloxacin against pathogenic *S. suis* isolates that had disseminated systemically from the respiratory tract to the brain, and a comparison of the two phases.

## 2. Materials and Methods

### 2.1. Initial Phase

A total of 635 isolates (*B. bronchiseptica* (116), *G. parasuis* (110), *P. multocida* (118), *S. suis* (254), and *M. hyopneumoniae* (37)) were previously obtained from 1340 animals during naturally occurring swine respiratory disease clinical trials. All procedures for media preparation, isolate preparation, and plate inoculation were performed using established protocols.

The MIC of pradofloxacin for each isolate was determined using a broth microdilution method in accordance with the recommendations presented by the Clinical and Laboratory Standards Institute (CLSI) [23]. At the time of this study, there were no recommendations for testing *G. parasuis* by CLSI, although experience indicated that MH-F broth worked for a majority of the clinical isolates. Additionally, there are no CLSI recommendations for *M. hyopneumoniae*; however, the procedures used were validated. The procedures and results for *G. parasuis* and *M. hyopneumoniae* were verified and validated at three separate commercial laboratories following standard operating procedures that were regulatory-compliant. Methods for determining *M. hyopneumoniae* antimicrobial susceptibility are included in the Supplementary Materials. The pradofloxacin used for this study had a potency of 996 µg/mg. The final pradofloxacin doubling dilution concentrations, after inoculation, ranged from 0.00013 to 32 µg/mL. The MIC plates were stored at ≤−65 °C and were considered stable for up to three months. Each plate included quality control (QC) tests. *Escherichia coli* (ATCC 25922), *Enterococcus faecalis* (ATCC 29212), *Pseudomonas aeruginosa* (ATCC 27853), *Staphylococcus aureus* (ATCC 29213), *Actinobacillus pleuropneumoniae* (ATCC 27090), *Histophilus somni* (ATCC 700025), *Streptococcus pneumoniae* (ATCC 49619), and *M. hyopneumoniae* (ATCC 25934) were used to verify the accuracy of the custom panel and the performance of the MIC tests. MIC results for QC organisms were within the acceptable ranges for all plates used in this study. Each plate also included positive and negative control wells. Positive control wells contained growth media with no antibiotics. Negative control wells were uninoculated growth media to ensure sterility. A test was considered valid only if the expected results were observed in the positive and negative control wells. Additionally, confirmation checks were performed on each isolate to ensure pure culture in the inoculum, proper growth in the MIC panel, and proper inoculum concentration.

The MIC results were interpreted as the lowest concentration of antimicrobial agent that completely inhibited the growth of the organism as detected by the growth or color change in the broth in the positive control well.

### 2.2. Second Phase

Thirty well-characterized serotype and multilocus sequence type (MLST) *S. suis* isolates were obtained from twenty-four swine farms across eleven U.S. states. The submitting veterinarian provided the sample type, age of the pig, and the U.S. state in which the farm was located. The isolates were recovered from pigs aged 2–11 weeks with a documented history of meningitis. To ensure genetic diversity, isolates were sourced from different farms or, if from the same farm, were recovered at least 3 months apart. The selection of diverse isolates from multiple geographic locations enhanced the representation of the study population and allowed for broader conclusions regarding pradofloxacin susceptibility. All procedures for media preparation, isolation, serotyping, and MLST were performed according to the established protocols of the Iowa State University (ISU) Veterinary Diagnostic Laboratory, Ames, IA 50011-1100, USA. The information collected on each isolate is detailed in Table 1.

The MIC of pradofloxacin for each isolate was determined using a broth microdilution method in accordance with the recommendations presented by the Clinical and Laboratory Standards Institute (CLSI) [24,25]. Customized 96-well Sensititre™ plates (Thermo Fischer Scientific™, West Sussex, UK) were used, containing a predetermined range of pradofloxacin concentrations (0.00012–8 µg/mL) in each well. Up to four isolates were tested per plate. All procedures for media preparation, isolate preparation, and plate inoculation were performed according to the manufacturer’s instructions and the established protocols of the ISU Veterinary Diagnostic Laboratory.

Each plate included QC tests. Initially, *Enterococcus faecalis* (ATCC 29212) and *Escherichia coli* (ATCC 25922) were used to verify the accuracy of the custom panel, following ISU’s internal QC procedures. The MIC results for both QC organisms were within the acceptable ranges for all plates used in this study. Each plate also included positive and negative control wells. A test was considered valid only if the expected results were observed in the positive and negative control wells. Additionally, confirmation checks were performed on the positive control well for each isolate. After AST was complete, a sample of the positive control well was streaked onto the appropriate agar plate (e.g., blood agar) and incubated, and growth was observed for purity to ensure pure culture in the inoculum.

The results of the sensitivity tests are presented as MIC distributions, and these were determined for each isolate. MIC_50_ and MIC_90_ were defined as MICs inhibiting 50% and 90% of the strains, respectively, while MIC_mode_ is the MIC value appearing the greatest number of times in the results.

## 3. Results

The pradofloxacin MIC results (cumulative) for *B. bronchiseptica*, *G. parasuis*, *M. hyopmoniae*, *P. multocida*, and *S. suis*, including minimum and maximum MICs (MIC range), MIC_mode_, MIC_50_, and MIC_90_, are summarized in Table 2.

The MIC results of the second-phase brain *S. suis* isolates were within the range of those in the initial phase. The MIC_mode_ and MIC_50_ were identical; however, the MIC_90_ was one doubling dilution less for the isolates originating in the brain.

## 4. Discussion

The different origins of isolates in each study phase resulted in slightly different MIC, MIC_mode_, MIC_50_, and MIC_90_ results. This comparison provides insights into pradofloxacin’s efficacy across different infection sites and stages of *S. suis* infection in swine. Furthermore, this research contributes to the broader understanding of antimicrobial resistance dynamics in veterinary medicine, and informs strategies for prudent antibiotic use in swine production systems.

This study encompassed two phases: the initial phase evaluated isolates originating from pleural swabs or lungs, which could either serve as a commensal habitat or a reservoir for pathogenic strains. In the initial phase, we observed that the pradofloxacin MICs for *S. suis* ranged from 0.015 to 8 µg/mL, with only two isolates (0.8%) at the upper MIC value of 8 µg/mL. The MIC_mode_, MIC_50_, and MIC_90_ values were 0.06 µg/mL and 0.25 µg/mL, respectively. Detailed MIC data for other swine respiratory pathogens, such as *B. bronchiseptica*, *G. parasuis*, *M. hyopneumoniae*, and *P. multocida*, were also recorded, with pradofloxacin demonstrating effective in vitro antimicrobial activity across these pathogens. The findings suggested that pradofloxacin has a broad spectrum of activity and may be effective against multiple swine respiratory pathogens.

In the second phase, we specifically selected pathogenic *S. suis* strains that had become systemic, migrating from the respiratory tract to the brain to cause meningitis. This selection criterion aimed to ensure that the isolates were representative of severe, invasive infections. The MIC values for the 30 *S. suis* isolates ranged from 0.015 µg/mL to 0.12 µg/mL, underscoring the variability in susceptibility among the *S. suis* isolates tested. The MIC_50_, which represents the concentration that inhibits 50% of the isolates, and the MIC_90_, which inhibits 90% of the isolates, were found to be 0.06 µg/mL and 0.12 µg/mL, respectively. This suggests that a majority of the isolates tested (90%) were inhibited by a relatively low concentration of pradofloxacin. The low MIC values witnessed in this study emphasize pradofloxacin’s potential as a viable treatment option for *S. suis* infections in swine.

Interestingly, the pradofloxacin MIC results for these pathogenic isolates closely mirrored those of the initial phase. While the MIC_mode_ and MIC_50_ were identical, the MIC_90_ was one doubling dilution less for the isolates originating in the brain (0.25 µg/mL versus 0.12 µg/mL). It may be hypothesized that antibiotic susceptibility is a factor of *S. suis* genomic differences between non-clinical commensal and clinical pathogenic (respiratory and systemic) isolates [26]. Systemic clinical isolates of *S. suis* typically have smaller genome sizes compared to respiratory and non-clinical isolates, despite harboring a greater number of virulence factors. In contrast, commensal isolates may carry more antimicrobial resistance (AMR) genes compared to systemic clinical isolates [26].

Although further clinical substantiation is needed, these findings are encouraging and indicate that pradofloxacin has the potential to be an effective therapeutic option for *S. suis* systemic infections in swine. A comparison with previous studies for other fluoroquinolones can shed light on potential trends in *S. suis* susceptibility [27,28]. In 2007, forty *S. suis* isolates from septicemic pigs in Germany, Belgium, and France demonstrated enrofloxacin MICs ranging from 0.12 to 2.0 µg/mL with a MIC_50_ of 0.25 µg/mL; ciprofloxacin (a metabolite of enrofloxacin) MICs ranging from 0.12 to 4.0 µg/mL with a MIC_50_ of 0.5 µg/mL; marbofloxacin MICs ranging from 0.25 to 2.0 µg/mL with a MIC_50_ of 0.5 µg/mL; danofloxacin MICs ranging from 0.25 to 1.0 µg/mL with a MIC_50_ of 0.5 µg/mL; difloxacin MICs ranging from 0.5 to 4.0 µg/mL with a MIC_50_ of 1.0 µg/mL; and norfloxacin MICs ranging from 1.0 to 8.0 µg/mL with a MIC_50_ of 2.0 µg/mL [27]. In 2023, another study of 29 *S. suis* isolates obtained from the nasal mucosa of asymptomatic pigs was conducted, and others were obtained from the lungs, joints, and abdomens of diseased pigs in Heilongjiang Province, China. The values are quite different, possibly due to the clinical history of the isolates, compared to the prior study values for enrofloxacin with MIC_50_ 8 µg/mL and MIC_90_ 64 µg/mL; ciprofloxacin at a MIC_50_ 4 µg/mL and MIC_90_ > 128 µg/mL; ofloxacin MIC_50_ 4 µg/mL and MIC_90_ 32 µg/mL; and dafloxacin with MIC_50_ 4 µg/mL and MIC_90_ 32 µg/mL [28]. These prior studies reported varying MIC ranges for other fluoroquinolones against *S. suis* isolates [27,28]. Although we cannot make a direct comparison, as there most likely are differences in the isolates between these two studies and the isolates described herein, pradofloxacin demonstrated a lower MIC range in our study population, suggesting potentially better in vitro activity.

However, it is crucial to consider, in addition to the MIC, pharmacokinetics (PK), pharmacodynamic (PD), and dosing regimens when evaluating the clinical utility of pradofloxacin in the treatment of *S. suis* infections in veterinary practice. Distinct from other fluoroquinolones, pradofloxacin, Pradalex, has unique PK/PD properties, with a higher maximum concentration (C_max_) of 2.5 µg/mL, a shorter time to reach maximum concentration (T_max_), 45 min, and a half-life (T_1/2_) of 8.2 h [29]. These properties are crucial for achieving and maintaining drug concentrations above the MIC for sufficient durations to inhibit bacterial growth effectively and limit the selection of resistant mutants.

Andes and Craig (2002) explored the PK/PD parameters of fluoroquinolones in experimental models of infection, highlighting the importance of C_max_ and T_1/2_ in achieving effective bacterial eradication [30]. Their work underscores the relevance of these parameters in the context of pradofloxacin’s efficacy. Additionally, Boerlin and White (2013) provide an overview of antimicrobial therapy in veterinary medicine, discussing the PK/PD properties of various drugs, including pradofloxacin, and their implications for resistance management. Their comprehensive examination of pradofloxacin’s pharmacological profile reinforces its strategic advantage in minimizing resistance [31].

Hence, our study demonstrates that pradofloxacin exhibits potent in vitro activity against *S. suis* and other key respiratory pathogens in swine. The MIC values only slightly differed between isolates originating from the respiratory tract versus the brain. This highlights its potential utility in managing bacterial infections in swine. To date, the specific concentration of pradofloxacin in the brain or central nervous system (CNS) of swine has not been investigated. Fluoroquinolones in general, because of their physiochemical properties, readily penetrate the CNS in the absence of meningeal inflammation [32]. This work provides a foundation for further investigations into pradofloxacin’s clinical applications and supports its consideration as a viable alternative in antimicrobial therapy for swine respiratory diseases, in general, and those that may penetrate the CNS.

Antimicrobial resistance is monitored globally to protect One Health [33,34]. The emergence of antimicrobial resistance in *S. suis* is a public health concern due to its zoonotic potential [35]. The findings of this study contribute to the growing body of knowledge on the susceptibility profiles of *S. suis* isolates to various antimicrobials. This information can be used to develop effective treatment strategies for swine infections and ultimately reduce the risk of the zoonotic transmission of *S. suis* to humans. Continuous monitoring of pradofloxacin susceptibility in *S. suis* isolates could help identify any emerging resistance trends and inform responsible antimicrobial stewardship practices in swine production to minimize the development of resistance.

## Figures and Tables

**Table 1:** Second-phase *Streptococcus suis* isolate demographics sorted by state.

Isolate ID	U.S. State *	Age (Week)	Type of Sample	Serotype	MLST Based Sequence Type
2022008942-1	AR	4	Brain Swab	17	778
2022008942-3	IA	4	Brain	- **	977
2022008942-4	IA	5	Brain	7	32
2022008942-6	IA	7	Brain	4	977
2022008942-7	IA	≤11	Brain	7	29
2022008942-8	IA	10	Brain	2	28
2022008942-12	IA	10	Brain	2	1
2022008942-13	IA	4	Brain	1	1
2022008942-14	IA	5	Brain	10	1170
2022008942-17	IA	4	Brain	7	373
2022008942-25	IA	5.7	Brain	3	108
2022008942-11	IL	10	Brain	24	94
2022008942-15	IL	6	Brain	33	1381
2022008942-16	IL	2.7	Brain	23	108
2022008942-5	IN	5	Brain	1	1
2022008942-19	IN	8	Brain	7	108
2022008942-26	IN	5	Brain Swab	5	977
2022008942-9	MI	5	Brain	4	485
2022008942-29	MI	2	Brain	1	1
2022008942-20	MN	6	Brain	3	94
2022008942-22	MN	2.3	Brain Swab	1	1
2022008942-24	MN	6	Brain	5	977
2022008942-23	MO	3	Brain	17	977
2022008942-30	MO	4	Brain	1	1
2022008942-18	OH	5	Brain	5	977
2022008942-27	OH	7	Brain	23	108
2022008942-2	OK	2.8	Brain	1	1
2022008942-28	PA	3	Brain	1/2	28
2022008942-10	SD	4	Brain	1/2	28
2022008942-21	SD	7	Brain	4	977

* United States of America state abbreviations: Arkansas (AR), Iowa (IA), Illinois (IL), Indiana (IN), Michigan (MI), Minnesota (MN), Missouri (MO), Ohio (OH), Oklahoma (OK), Pennsylvania (PA), South Dakota (SD). ** Isolate did not react to any of the antisera tested. This isolate’s serotype is unknown.

**Table 2:** MIC of pradofloxacin for *Bordetella bronchiseptica*, *Glaesserella parasuis*, *Mycoplasma hyopneumoniae*, *Pasteurella multocida*, and *Streptococcus suis*.

Pathogens *	Origin of Sample	No. of Isolates	MIC_mode_	MIC_50_ ** (μg/mL)	MIC_90_ ** (μg/mL)	MIC Range (μg/mL)
*B. bronchiseptica*	Lung/Pleural Swab	116	0.12	0.12	0.12	0.12–0.25
*G. parasuis*	Lung/Pleural Swab	119	0.002	0.002	0.004	0.00025–0.008
*P. multocida*	Lung/Pleural Swab	118	0.004	0.004	0.008	0.004–0.015
*M. hyopneumoniae*	Lung/Pleural Swab	37	0.004	0.004	0.008	0.00013–0.015
*S. suis*	Lung/Pleural Swab	254	0.06	0.06	0.25	0.015–8
*S. suis*	Brain/Brain Swab	30	0.06	0.06	0.12	0.015–0.12

* The correlation between in vitro susceptibility data and clinical effectiveness is unknown. ** The lowest MIC to encompass 50% and 90% of the most susceptible isolates, respectively.

## Data Availability

The original contributions presented in this study are included in the article/Supplementary Materials. Further inquiries can be directed to the corresponding author.

## Supplementary Materials

The following supporting information can be downloaded at https://www.mdpi.com/article/10.3390/pathogens14010088/s1, Protocol S1: *Mycoplasma hyopneumoniae* susceptibility testing.
