# Supplementary material for: Pradofloxacin Minimum Inhibitory Concentration Profiling of Streptococcus suis Isolates: Insights into Antimicrobial Susceptibility in Swine
Source: Pathogens. 2025 Jan 17;14(1):88. doi: 10.3390/pathogens14010088 (PMC11768269; doi:10.3390/pathogens14010088)
Supplement: Supplementary file 1 [file pathogens-14-00088-s001.zip › pathogens-3396739-supplementary.pdf]

Protocol S1. *Mycoplasma hyopneumoniae* Susceptibility Testing

Section 1: Culture Media

Modified Hanks Balanced Salt Solution (HBSS)

Part A:

NaCl	80 g
KCl	4 g
$MgSO_4 \cdot 7H_2O$	1 g
$MgCl_2 \cdot 6H_2O$	1 g

Dissolve the listed ingredients in 350-450 mL of sterile deionized water. Then add 1.4 g $CaCl_2$. Mix until dissolved and q.s. to 500 mL with sterile deionized water. Filter sterilize through a 0.22 µm or 0.45 µm filter.

Store in a tightly sealed container at 2-8°C for up to 3 months or indefinitely at ≤ -65°C.

Part B:

$Na_2HPO_4 \cdot 12H_2O$ 1.5 g

Dissolve in 350-450 mL sterile deionized water. Then add 0.6 g KH_2PO_4. Mix until dissolved and q.s. to 500 mL with sterile deionized water. Filter sterilize through a 0.22 µm or 0.45 µm filter.

Store in a tightly sealed container at 2-8°C for up to 3 months or indefinitely at ≤ -65°C.

Yeast Extract

Add 125 g dry *Saccharomyces cerevisiae* (active dry yeast, e.g., Fleischmann's) to 750 mL of sterile deionized water. Adjust amounts if a different volume is desired. Mix to suspend and then place in a 36-38°C water bath. Monitor the temperature of the solution, and when reaches ≥ 35°C, time for 20 minutes, mixing frequently. Then immediately place in a boiling (90-100°C) water bath. Monitor the temperature of the solution and when reaches ≥ 60°C, time for 5 minutes. Remove and let cool. Centrifuge at 3000xG for 30 minutes avoid stirring up the pellet.

Remove supernatant fluid and filter through a Whatman #1 filter. Sterilize at 15 lb pressure for 15 minutes in pressure cooker. Additional time may be required for complete sterilization depending upon the volume. Cool then dispense into sterile containers with sterile lids. Expiration is indefinite if stored at ≤ -65°C or 3 months if stored at ≤ -18°C.

Note: There may be precipitation when the extract is removed from the autoclave or pressure cooker. If desired, aseptically filter through sterile cheese cloth or other filter material (e.g., sterile coffee filters) to remove remaining sediment.

Phenol Red Solution (0.5%)

Grind 2.5 g of phenol red in a mortar with successive additions of 0.1N NaOH until 75 mL has been added. Bring to 500 mL with deionized water and refrigerate (2-8°C) overnight. Filter through a #1 Whatman or similar filter. Adjust pH to 7.0±0.1. Dispense in desired amounts in a tightly sealed container. Sterilize at 15 lb pressure for 15 minutes in a pressure cooker. Store at ≤-65°C.

FRIIS Broth:

Combine: (adjust amounts if another volume is desired)

HBSS - Part A	40 mL
HBSS - Part B	40 mL

Add to 700 mL of deionized water (≥ 17.5 µmhos resistivity) and mix; then add the following ingredients and mix, without heat, to dissolve.

Brain Heart Infusion Broth (e.g., Difco)	5.0 g
PPLO Broth (e.g., Difco)	5.2 g

Add remaining deionized water (1170 mL of water total)	470 mL

Sterilize at 15 lb pressure for 15 minutes in pressure cooker. Cool to room temperature and add the following sterile solutions.

Yeast Extract	60 mL
Phenol Red (0.5% solution)	4.5 mL
Horse Serum (heat inactivated)	165 mL
Swine Serum (heat inactivated)	165 mL

Mix 2-3 minutes. If necessary to adjust pH, use HCl or NaOH solutions. Final pH of media should be 7.5-7.6 (light red or salmon color). Test tube caps should be tightly sealed to prevent color change during storage. Media may be stored indefinitely at ≤ -65°C.

Section 2: Preparation of Inoculum

Grow the *Mycoplasma* isolates in 2±1 mL of FRIIS broth aerobically at 36±2°C preferably with constant movement (e.g., roller drum or shaker). Once grown use a 10% inoculum of

the grown broth to pass into fresh FRIIS broth and incubate until just grown. Overgrowth of the culture should be avoided.

The use of phenol red indicator should be interpreted as a color change from red to yellow/orange. Incubation to yellow may be too long and should be avoided. The color change described indicates an approximate concentration of 10^8 color changing units (CCU)/mL.

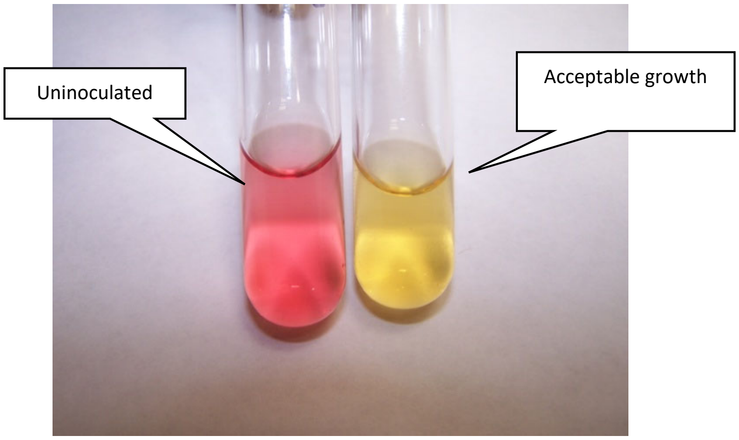

Section 3: Inoculation and Interpretation of MIC

1. Thaw required number of MIC plates if prepared ahead of time. MIC plates for *Mycoplasma hyopneumoniae* must be filled with 50 µL of antimicrobial dilution that is 2 times the intended final concentration. The growth control well must be filled with 100 µL of FRIIS broth.

2. Dilute 4 µL of the grown culture in 6 mL of FRIIS broth. The volume of media may be changed, although the amount of inoculum must remain proportional unless colony counts indicate the inoculum amount should be changed to achieve the desired final inoculum level.

3. Mix by vortexing, pour into an inoculum tray and inoculate wells (including turbidity or growth control well) of a labeled MIC plate with 50 µL of diluted, standardized culture, except for the sterility well. No inoculated broth will be added to the sterility well. The final estimated concentration of *Mycoplasma* in the MIC plate theoretically should be 10^4 to 10^5 CCU/mL.

4. Apply an individual adhesive seal to each MIC plate to prevent dehydration of the broth. Incubate the plates aerobically at 36±2°C until a color change occurs in the growth control well (up to 14 days). Compare the color change or turbidity of the antibiotic containing wells to the growth and sterility control wells of the MIC plate to determine the MIC.
